# Factors associated with unprotected anal sex with multiple non-steady partners in the past 12 months: results from the European Men-Who-Have-Sex-With-Men Internet Survey (EMIS 2010)

**DOI:** 10.1186/s12889-016-2691-z

**Published:** 2016-01-19

**Authors:** Sarah C. Kramer, Axel Jeremias Schmidt, Rigmor C. Berg, Martina Furegato, Harm Hospers, Cinta Folch, Ulrich Marcus, for the EMIS Network

**Affiliations:** 1 Department of Infectious Disease Epidemiology, Robert Koch Institute, Berlin, Germany; 2 Sigma Research, Department of Social and Environmental Health Research, London School of Hygiene & Tropical Medicine, 15-17 Tavistock Place, WC1H 9SH London, UK; 3 Department of Evidence-Based Health Services, Norwegian Knowledge Center for the Health Services, Oslo, Norway; 4 HIV & STI Department, Centre for Infectious Disease Surveillance and Control, Public Health England, London, UK; 5 University College Maastricht, Maastricht University, Maastricht, The Netherlands; 6 Centre for Sexually Transmitted Infection and AIDS Epidemiological Studies of Catalonia (CEEISCAT), Barcelona, Spain

## Abstract

**Background:**

Practising unprotected anal intercourse (UAI) with high numbers of partners is associated with increased risk for acquiring and transmitting HIV and other sexually transmitted infections. Our aim was to describe factors associated with UAI with multiple partners in a large sample of MSM from 38 European countries recruited for an online survey in 2010.

**Methods:**

Data are from the European Men-Who-Have-Sex-With-Men Internet Survey (EMIS). The analysis was restricted to men who reported any anal sex with a non-steady partner in the past 12 months, and who were either never diagnosed with HIV, or who had been diagnosed with HIV more than 12 months ago, reported a detectable viral load and did not exclusively serosort (*n* = 91,477). Multivariable logistic regression was used to compare men reporting UAI with four or more (4+) non-steady partners to two comparison groups: a) no UAI with non-steady partners, and b) UAI with 1-3 non-steady partners.

**Results:**

Overall, 9.6 % of the study population reported UAI with 4+ partners in the past 12 months. In both models, factors consistently associated with this behaviour were: having been diagnosed with HIV, lower educational levels, use of nitrite inhalants, drugs associated with sex and parties, or erectile dysfunction drugs in the past 4 weeks, using sex-on-site venues in the past 4 weeks, buying or selling sex in the past 12 months, having experienced physical violence due to sexual attraction to men in the past 12 months, reporting sexual happiness, being out to all or almost all of one’s acquaintances, and knowing that ART reduces HIV transmissibility.

**Conclusions:**

Effective antiretroviral treatment drastically reduces HIV transmission for men diagnosed with HIV, irrespective of partner numbers. Apart from reducing partner numbers or increasing condom use no other recommendations are currently in place to reduce the risk of HIV acquisition and onward transmission for HIV-negative men practicing UAI with multiple partners. A range of factors were identified as associated with UAI with four or more partners which allow the strengthening and targeting of prevention strategies to reduce HIV transmission risks resulting from condomless anal intercourse with multiple partners.

## Background

Throughout Europe, men who have sex with men (MSM) continue to be disproportionately affected by the HIV epidemic [1], with self-reported HIV prevalences ranging from 0 to 20.0 %, and measured HIV prevalences ranging from 0.7 to 17.0 % [2]. However, these numbers are likely to be inflated due to (self-) selection biases in the respective studies, while on the other hand self-reported prevalence underestimates HIV prevalence because it only includes diagnosed infections. Unprotected anal intercourse (UAI) is the main transmission mode among MSM [3]. The risk of HIV transmission during anal sex can be reduced by proper condom use, HIV serosorting based on correct and up-to-date HIV serostatus knowledge of all involved partners, effective antiretroviral treatment (ART) of the HIV-infected partner, or oral chemoprophylaxis (also known as pre-exposure prophylaxis or PrEP) for the non-HIV-infected partner [4]. Other risk reduction tactics and strategies like strategic positioning, withdrawal from penetrative intercourse before ejaculation, restricting UAI to a steady partner, and HIV serostatus disclosure in order to forgo condom use are of variable or questionable efficacy, and depend on accurate knowledge of one’s own and of the partners’ serostatus [5]. Since the majority of MSM in Europe have been tested for HIV at least once [2, 6, 7] and intentional transmission or acquisition of HIV are extremely rare, most new transmissions occur when HIV serostatus is not or is incorrectly communicated between sex partners. The latter may involve people making incorrect assumptions about serostatus concordance without direct communication, being unaware of having been infected since the last negative HIV test, or communicating an outdated negative serostatus. For example, only about half of EMIS respondents reporting UAI in the past 12 months had also been tested for HIV in the same time frame [6]. It is not uncommon that men engage in a number of episodes of UAI with multiple partners before they re-assess their HIV status. In addition, the more partners with whom an individual engages in UAI, the more difficult it becomes to be sure of one’s status. Modern HIV tests (fourth generation) can reliably exclude HIV infection by approximately 6 weeks after exposure [8]; however, rare cases of seroconversion have been reported up to 90 days after exposure [9]. Thus, if an HIV-negative individual has UAI with four or more (4+) partners per year, it may become challenging to manage HIV transmission risks by correct serostatus knowledge.

In this study, we perform exploratory analyses on a large, cross-sectional dataset of MSM from 38 European countries in order to highlight the factors associated with men having UAI with 4+ non-steady partners in the past 12 months (in other words, on average at least one different partner with whom UAI was practiced every 3 months), as compared to men engaging in UAI with fewer non-steady partners or consistently using a condom. The terminology UAI is used here to describe what was queried as “anal intercourse without a condom”, without the use of antiretroviral treatment or oral chemoprophylaxis, and without knowledge about HIV positive seroconcordance between partners. We examine primarily individual-level factors, but also assess the extent to which country of residence explains variation in response among individuals. While engaging in UAI with multiple partners is certainly not the only way for MSM to acquire and transmit HIV, the subgroup of MSM with multiple UAI partners is theoretically at high risk of acquiring and/or transmitting HIV. Therefore, this subgroup is particularly important for the dynamics of the HIV epidemic among MSM. It is also a subgroup which, from a public health standpoint, could benefit most from oral chemoprophylaxis – if not yet infected with HIV. In this analysis we describe and characterize a subgroup of MSM at increased risk for HIV acquisition and transmission due to UAI with multiple non-steady partners; we do not report on transmission risks within steady partnerships.

## Methods

### Data source

We used data from the 2010 European Men-Who-Have-Sex-With-Men Internet Survey (EMIS). Participants were recruited primarily from dating and other social networking websites specifically targeting MSM. The detailed methods of EMIS have been reported elsewhere [10]. In brief, EMIS was an anonymous, self-administered online survey conducted simultaneously in 25 languages across 38 countries, with a final sample size of 174,209 respondents. Typical completion time was 20 min (calculated from the precise completion time for each survey, auto-captured by the survey software). No financial incentives were given. No IP addresses were collected. The survey was accessible online from June 6 to August 31, 2010. Most participants were recruited on five international commercial websites by instant messages. On the largest recruiter website response rates per country ranged from 4.4 to 15 % of those who had been targeted by instant messages. The mean submission rate of evaluable questionnaires across the 25 language versions among those who went beyond the introduction page was 68.5 % (range 62 % to 76 %). Differences in the survey methods among the 38 countries are described in detail elsewhere [6, 10]. Potential sampling biases and representativeness of the samples have been analysed in three different publications [2, 11, 12]. More background information, including the English version of the questionnaire, is available at www.emis-project.eu and in [6].

Ethical approval for EMIS was given by the Research Ethics Committee of the University of Portsmouth, UK (REC application number 08/09:21).

### Analytic sample

**Fig. 1:**
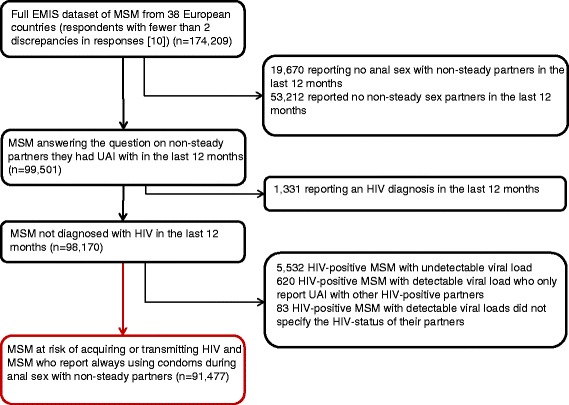
Selection of the analytic sample

For our analyses, the dataset was restricted to MSM reporting anal sex with at least one non-steady male sex partner in the past 12 months. Men who did not answer the subsequent question on the number of partners they had UAI with were excluded. Additionally, we removed men who had been diagnosed with HIV in the past 12 months, as it was not possible in these cases to determine whether sexual risks reported in the last 12 months occurred before or after HIV diagnosis. Finally, we removed HIV-diagnosed men reporting undetectable viral load, as these men are unlikely to transmit HIV to uninfected partners [13]. For the same reason, HIV-diagnosed men who reported only engaging in UAI with other HIV diagnosed men were also removed from analysis. This process is summarized in Fig. 1. The resulting dataset consisted of 91,477men.

### Dependent variable

We split the remaining dataset into three groups, based on the number of non-steady male partners with whom UAI was reported in the past 12 months (none - i.e. only protected anal intercourse with non-steady partners, one to three (1-3), 4+). Using two comparison groups allowed us to not only observe differences between men with many and few non-steady partners, but also to examine whether the impact of various factors increases as the number of partners rises.

### Independent variables

A variety of potential independent variables were included in the models, including socio-demographics such as age, settlement size, education (assessed with the 1997 version of the 6-level International Standard Classification of Education, ISCED), occupation, country of birth, sexual identity, relationship status, and outness; behaviour (any sex with women in the past 12 months, use of venues where on-site sex is possible, buying or selling sex); drug use (nitrite inhalants; drugs associated with sex and parties such as ecstasy, amphetamines, crystal methamphetamine, mephedrone, GHB, ketamine, and cocaine; erectile dysfunction drugs); psychological variables (sexual happiness, loneliness); discrimination (experiencing violence due to sexual attraction to men); and HIV-related knowledge (knowing that antiretroviral treatment (ART) reduces HIV transmissibility, being exposed to HIV-related information for MSM in the past 12 months). These variables were chosen prior to initiation of analyses, and decisions were based on a literature review. Additionally, we control for HIV-diagnosis. All variables were self-reported. Participants’ use of on-site sex venues and drugs were assessed in the past 4 weeks; violence and transactional sex were assessed in the past 12 months. Loneliness was dichotomized based on whether participants agreed or strongly agreed with the statement “I sometimes feel lonely.”

### Statistical analyses

Candidate variables to include in the final multivariable regression models were selected using univariable logistic regression and multivariable logistic regression controlling for HIV diagnosis.

For each of the three multivariable models (one for each reference group compared with the 4+ group, and one to compare the 1-3 UAI partner group with the none UAI partner group), potential predictor variables were considered progressively in two groups. First, all socio-demographic variables (with the exception of outness, which was included in step 2) found to be significant after controlling for HIV diagnosis were entered into the model, then assessed for retention [14] using chi-square tests to compare model fit when individual variables were dropped. When no more variables could be dropped, all other variables significant after controlling for HIV diagnosis were progressively considered for inclusion in the model. Variables were reconsidered for dropping at each step. Due to the low number of HIV-diagnosed men in our analytic sample, we were unable to assess interactions between independent variables and HIV-status. Potential interactions between other variables were identified after all main effects were included, and were assessed for relevance. Final fixed effects models were assessed using cross-validation and visual inspection of residuals and cooks distances. Because of the size of our dataset and due to the number of potential variables included, variables were only considered to be significant at the *p* = 0.01 level. Because the data had two levels, the country level and individual level, multilevel modelling was used.

## Results

### Descriptive statistics (country-level)

**Fig. 2:**
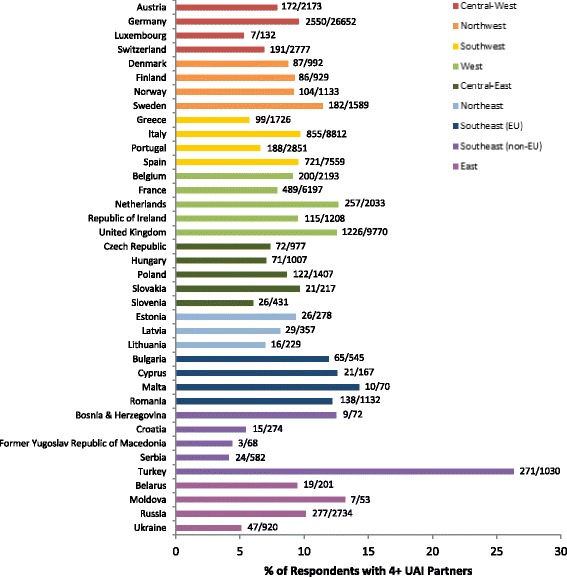
Proportion of respondents reporting 4+ partners from each country. Bars indicate the percentage of respondents reporting UAI with four or more non-steady male partners in the past 12 months; the number of respondents reporting UAI with 4+ non-steady partners over the total number of respondents from the country is displayed to the right of each bar. The region to which each country belongs is indicated by the colour of the bars

Our analytic sample contains information on 91,477 men from 38 different countries. The number of participants from each country surveyed, as well as the percentage of respondents reporting UAI with 4+ partners broken down by country, is shown in Fig. 2. As in the total EMIS sample [6], the largest country sample was from Germany (*n* = 26,652), with the United Kingdom in a distant second (*n* = 9770). The proportion of respondents reporting UAI with 4+ non-steady partners in the last 12 months ranged from 4.1 % (Serbia) to 26.3 % (Turkey), with no clear regional pattern.

### Descriptive statistics (individual-level)

Of 91,477 respondents in the analytic sample, 50,809 (55.6 %) reported no UAI with non-steady partners in the past 12 months, 31,850 (34.8 %) reported UAI with one to three partners, and 8818 (9.6 %) with four or more. This number corresponds to 5.1 % of the total EMIS sample (*N* = 174,209). Descriptive statistics are shown in Table 1. Briefly, about one-fifth (22.1 %) of respondents were under the age of 25, and slightly more (26.4 %) were 40 or older. Almost two-thirds (74.8 %) of participants had post-secondary education (ISCED >3), and 25.9 % had obtained advanced research qualification (ISCED = 6). A sizeable proportion (14.9 %) of participants was born abroad. The majority of participants (77.7 %) identified as homosexual, and 39.4 % overall reported a steady partner, either male or female. The remaining number of participants with longstanding HIV diagnosis but non-effective treatment or no treatment at all was small (2,445, 2.7 %). About a quarter of men had never tested for HIV (*n* = 22,938, 25.1 %).

**Table 1:** Descriptive statistics of the study sample by reported number of non-steady male UAI partners

Number of UAI Partners	0 (*n* = 50,809)	1–3 (*n* = 31,850)	4+ (*n* = 8818)
Age			
< 25	10,152 (20.0 %)	8434 (26.5 %)	1662 (18.9 %)
25–39	26,962 (53.1 %)	15,672 (49.2 %)	4438 (50.3 %)
40+	13,695 (26.9 %)	7744 (24.3 %)	2718 (30.8 %)
Settlement size: 500,000 inhabitants or more	24,624 (48.5 %)	14,040 (44.1 %)	4423 (50.2 %)
Education°			
ISCED 1–3	11,059 (21.8 %)	8908 (28.1 %)	2625 (29.9 %)
ISCED 4	10,558 (20.9 %)	7618 (24.0 %)	1972 (22.5 %)
ISCED 5	14,012 (27.7 %)	8390 (26.5 %)	2225 (25.4 %)
ISCED 6	14,969 (29.6 %)	6780 (21.4 %)	1946 (22.2 %)
Employed (including part-time and self-employment)	38,080 (74.9 %)	22,518 (70.7 %)	6603 (74.9 %)
Born abroad	7708 (15.2 %)	4433 (13.9 %)	1446 (16.4 %)
Sexual identity			
Gay/Homosexual	39,359 (77.6 %)	24,552 (77.3 %)	7164 (81.5 %)
Bisexual	7529 (14.9 %)	4727 (14.9 %)	1088 (12.4 %)
Other	3819 (7.5 %)	2483 (7.8 %)	540 (6.1 %)
In steady relationship with a man?	21,635 (42.6 %)	11,201 (35.2 %)	3245 (36.8 %)
Diagnosed with HIV	914 (1.8 %)	564 (1.8 %)	967 (11.0 %)

° ISCED = International Standard Classification of Education, 1997 version

### Univariable and other initial analyses

Results of univariable analyses were fairly consistent for both comparison groups. Specifically, men reporting UAI with 4+ non-steady partners tended to be older, to live in larger cities, to be less educated, to have been born abroad, to identify as homosexual, to be diagnosed with HIV, to be out to all or almost all of their acquaintances, to be more happy with their sex lives, to have experienced physical violence due to their sexual orientation during the past 12 months, to have used a gay sex venue during the past 4 weeks, to have bought or sold sex during the past year, to know that ART reduces HIV transmissibility, and to report using nitrite inhalants, drugs associated with sex and parties, or erectile dysfunction drugs in the past 4 weeks. Compared to men with no UAI, men engaging in UAI with 4+ non-steady partners were more likely to report feeling lonely and to have experienced intimidation or verbal abuse, and were less likely to be in a steady relationship, or to have been exposed to MSM-specific information about HIV or STIs in the past 12 months; the direction of these relationships was reversed when men reporting UAI with 1-3 non-steady partners were used as the reference group. Finally, compared to the group reporting UAI with 1-3 non-steady partners, men reporting UAI with 4+ non-steady partners were also more likely to be employed. Results of univariable analyses are presented in Table 2.

**Table 2:** Factors associated with UAI with multiple non-steady sexual partners. Odds ratios and 95 %-confidence intervals from univariable logistic regression models

	4+ vs. None	4+ vs. 1–3
Age		
< 25	0.82 (0.77–0.88)***	0.56 (0.52–0.60)***
25–39	0.83 (0.79–0.87)***	0.81 (0.76–0.85)***
40+	1.00 (ref)	1.00 (ref)
Settlement Size: 500,000 inhabitants or more	1.08 (1.03–1.13)**	1.30 (1.24–1.36)***
Education°		
ISCED 1–3	1.00 (ref)	1.00 (ref)
ISCED 4	0.79 (0.74–0.84)***	0.88 (0.82–0.94)**
ISCED 5	0.67 (0.63–0.71)***	0.90 (0.84–0.96)*
ISCED 6	0.55 (0.51–0.58)***	0.97 (0.91–1.04)
Employed	1.00 (0.95–1.05)	1.24 (1.17–1.31)***
Born abroad	1.09 (1.03–1.16)*	1.21 (1.13–1.29)***
Sexual Identity		
Gay/homosexual	1.00 (ref)	1.00 (ref)
Bisexual	0.79 (0.74–0.85)***	0.79 (0.73–0.85)***
Other	0.78 (0.71–0.85)***	0.75 (0.68–0.82)***
In Steady Relationship	0.78 (0.75–0.82)***	1.07 (1.02–1.13)*
Diagnosed HIV infection	6.72 (6.12–7.38)***	6.83 (6.14–7.61)***
Any sex with women in past 12 months	0.97 (0.90–1.03)	1.03 (0.96–1.10)
Out to everyone or almost everyone participant knows	1.34 (1.28–1.41)***	1.41 (1.34–1.48)***
Feelings of loneliness	1.17 (1.11–1.22)***	0.82 (0.78–0.86)***
Sexual happiness	1.24 (1.18–1.31)***	1.61 (1.53–1.69)***
Experience of violence due to sexual attraction to men in past 12 months		
Physical abuse	2.36 (2.09–2.65)***	1.72 (1.52–1.94)***
Intimidated or Verbal Abuse	1.10 (1.05–1.15)**	0.94 (0.90–0.99)^+^
None	1.00 (ref)	1.00 (ref)
Visits to gay sex venues in past 4 weeks	2.70 (2.58–2.83)***	2.80 (2.66–2.94)***
Bought sex in past 12 months	1.55 (1.44–1.66)***	1.54 (1.44–1.66)***
Sold sex in past 12 months	3.97 (3.70–4.25)***	2.47 (2.31–2.65)***
Exposed to information about HIV/STIs for MSM in past 12 months	0.85 (0.80–0.91)***	1.13 (1.06–1.20)**
Knew that effective treatment of HIV infection reduces the risk of HIV being transmitted	1.34 (1.28–1.40)***	1.49 (1.42–1.56)***
Use of nitrite inhalants in past 4 weeks	2.71 (2.59–2.84)***	2.50 (2.38–2.63)***
Use of drugs associated with sex and parties in past 4 weeks	3.26 (3.04–3.50)***	2.30 (2.14–2.47)***
Erectile dysfunction drugs in past 4 weeks	3.36 (3.16–3.57)***	2.77 (2.60–2.96)***

(^+^ *p* < 0.05, *p < 0.01, **p < 0.001, ***p < 0.0001)

° ISCED = International Standard Classification of Education, 1997 version

### Multivariable analyses

In both multivariable models comparing the 4+ group, the variables significantly and positively associated with reporting UAI with 4+ non-steady partners at the *p* < 0.01 level were: being diagnosed with HIV, reporting use of nitrite inhalants, drugs associated with sex and parties, or erectile dysfunction drugs in the past 4 weeks, having bought or sold sex in the past 12 months, visiting a sex-on-site venue in the past 4 weeks, reporting sexual happiness, being out to all or most acquaintances, having experienced physical violence due to sexual attraction to men in the past 12 months, and knowing that ART reduces HIV infectivity. Higher education was significantly and negatively associated with reporting UAI with 4+ non-steady partners in both models. Additionally, when compared to the non-UAI group, men in the 4+ group were more likely to sometimes feel lonely and to report intimidation or verbal violence due to sexual attraction to men in the past 12 months, and were less likely to be in a steady relationship, particularly one where they felt sexually happy, and to have been exposed to MSM-targeted information on HIV and STIs in the past 12 months. Compared to men engaging in UAI with 1-3 non-steady partners, men in the 4+ partner group were less likely to be under the age of 25. Results can be observed in Table 3.

**Table 3:** Factors associated with UAI with multiple non-steady sexual partners. Adjusted odds ratios and 99 %-confidence intervals from multivariable mixed-effects logistic regression models

	4+ vs. None	4+ vs. 1–3	1–3 vs. None
Diagnosed HIV infection	4.10 (3.54–4.75)***	3.96 (3.36–4.66)***	0.95 (0.82–1.11)
Age			
< 25	N.S.	0.77 (0.69–0.87)***	1.18 (1.10–1.26)***
25–39	N.S.	0.92 (0.85–1.01)^+^	1.00 (0.95–1.05)
40+	N.S.	1.00 (ref)	1.00 (ref)
Education°			
ISCED 1–3	1.00 (ref)	1.00 (ref)	1.00 (ref)
ISCED 4	0.85 (0.77–0.93)***	0.93 (0.84–1.03)	0.87 (0.82–0.93)***
ISCED 5	0.67 (0.61–0.74)***	0.88 (0.79–0.97)***	0.73 (0.69–0.77)***
ISCED 6	0.55 (0.50–0.61)***	0.85 (0.76–0.94)***	0.61 (0.57–0.65)***
Settlement size: 500,000+	N.S.	1.05 (0.97–1.13)	0.85 (0.81–0.88)***
Unemployed/Student/Retired/Other	N.S.	N.S.	1.06 (1.01–1.12)*
Sexual happiness	1.31 (1.20–1.44)***	1.38 (1.28–1.49)***	0.93 (0.88–0.98)**
In steady relationship		N.S.	
Happy w/ sex life	0.60 (0.55–0.66)***		0.77 (0.73–0.81)***
Not happy w/ sex life	0.84 (0.74–0.97)*		0.93 (0.86–0.997)*
Out to all or almost all people participant knows	1.13 (1.04–1.22)**	1.14 (1.06–1.23)***	N.S.
Feelings of loneliness	1.15 (1.07–1.24)***	N.S.	1.24 (1.18–1.29)***
Experience of violence due to sexual attraction to men in past 12 months			
Physical	1.67 (1.39–2.01)***	1.54 (1.28–1.87)***	1.07 (0.94–1.22)
Intimidation or verbal abuse	1.08 (1.00–1.16)*	1.00 (0.93–1.08)	1.09 (1.05–1.14)***
Visits to gay sex venues in past 4 weeks	2.19 (2.04–2.35)***	2.10 (1.95–2.26)***	N.S.
Sold sex in past 12 months	2.89 (2.59–3.22)***	2.13 (1.91–2.39)***	1.39 (1.27–1.51)***
Bought sex in past 12 months	1.38 (1.24–1.54)***	1.25 (1.12–1.40)***	N.S.
Exposed to information about HIV/STIs for MSM in past 12 months	0.76 (0.69–0.84)***	0.91 (0.82–1.00)^+^	0.83 (0.79–0.88)***
Knew that effective treatment of HIV infection reduces the risk of HIV being transmitted	1.14 (1.06–1.22)***	1.18 (1.09–1.27)***	N.S.
Use of nitrite inhalants in past 4 weeks	1.89 (1.75–2.05)***	1.60 (1.47–1.73)***	1.21 (1.15–1.28)***
Use of drugs associated with sex and parties in past 4 weeks	1.60 (1.42–1.79)***	1.22 (1.09–1.38)***	1.35 (1.24–1.46)***
Erectile dysfunction drugs in past 4 weeks	2.07 (1.87–2.28)***	1.65 (1.48–1.1.83)***	1.29 (1.19–1.39)***

N.S. indicates that the variable was not selected for inclusion in the model (^+^ *p* < 0.05, *p < 0.01, **p < 0.001, ***p < 0.0001)

° ISCED = International Standard Classification of Education, 1997 version

In the multivariable model comparing the 1-3 UAI partner group with the no UAI partner group, HIV diagnosis was not significant. Distinct from the 4+ group, factors positively associated with reporting UAI with 1-3 non-steady partners were age below 25 years, not being employed, and living in a place with less than 500,000 inhabitants. Contrary to the 4+ group, the 1-3 UAI partner group was sexually less happy. Outness, visits to gay sex venues, buying sex and knowing that effective treatment of HIV infection reduces the risk of HIV transmission were all not significant. Experience of physical violence was not significant, but experience of intimidation and verbal abuse was.

### Mixed effects models

Adding country as a random effect to the models comparing the 4+ group to the group with no UAI or UAI with 1-3 non-steady partners accounted for 22.0 % and 8.2 %, respectively, of the total variance in response. As expected based on the descriptive statistics, this was mostly due to the influence of Turkey. The random effect was retained in both models for consistency.

## Discussion

In this study, we analysed the responses of a large number of European MSM in order to determine what factors were associated with reporting UAI with four or more non-steady male partners in the past 12 months. Most factors identified as being significantly associated with UAI with four or more partners were fairly consistent across both multivariable models, with more and larger differences typically existing when men with fewer non-steady partners were taken to be the comparison group. Therefore, many risk factors in our models increased in impact with an increase in the number of non-steady male partners with whom a man engages in UAI. When comparing the group reporting UAI with 1-3 non-steady partners in the past 12 months with men not reporting UAI with non-steady partners it differs from the respective comparison of the 4+ group. The 1-3 UAI partner group consists of more younger men still in education and men living outside of the large cities having less access to gay sex venues.

Of note, with the exception of Turkey, the country of residence did not greatly impact the probability of reporting UAI with multiple non-steady partners, despite the wide range of legal and social attitudes toward homosexuality in the countries surveyed. However, as laws and social attitudes toward homosexuality are likely to influence some of the individual-level factors in our model, such as experience of physical violence or exposure to HIV-related knowledge, the indirect impact of oppressive laws and general discrimination should not be discounted. Turkey differs from the other countries in the sample by being the only major country with a Muslim majority (the other being Bosnia and Herzegovina, with only 72 respondents), and consequently a different cultural background. Stigmatization and discrimination of MSM in these two countries is high, but this is also the case for several other countries in the sample, e.g. Russia, Bulgaria, and Romania [15, 16]. Thus, we have no ready explanation for this finding, except that there is very little HIV prevention messaging for MSM in Turkey and a very weak “gay community”. Additional research will be necessary to explain this finding, particularly since HIV prevalence – although self-reported – is low in the Turkish sample (3 % among Turkish EMIS participants ever tested for HIV).

Even after exclusion of HIV-positive men with undetectable viral loads and those who consistently practice HIV-positive serosorting, men who reported a previous HIV diagnosis were significantly more likely to report UAI with 4+ partners than men who had never been diagnosed. This finding is consistent with previous research [17–19], and may reflect selection (e.g. use of gay dating sites for recruitment) and self-selection (e.g. men interrupting sexual activity after HIV diagnosis may select not to participate in such a survey) effects in our sample, a continuation of or a return to risky pre-infection sexual behaviour, or may be explained by a lack of perceived relevance of restriction of partner numbers as an HIV prevention strategy once HIV has been diagnosed. To mitigate the high levels of non-seroconcordant UAI reported by men diagnosed with HIV, it is necessary that prevention campaigns continue to target men diagnosed with HIV, and not only presumably uninfected men. As emphasized in the 2013 WHO HIV treatment guidelines [20], the prevention effects of early initiation of antiretroviral treatment should be maximised by prompt diagnosis followed by offering immediate and affordable treatment. Since the START trial provided evidence that early antiretroviral treatment is also beneficial for maintaining the health of the individual [21], public health and individual health benefits are not conflicting goals, which can be emphasized when discussing treatment options with newly diagnosed HIV patients. Policy makers should reconsider policies that recommend waiting until CD4 counts have dropped to a certain level before initiating treatment, and instead recommend treatment immediately upon diagnosis.

Despite previous evidence suggesting a protective effect of higher age [22, 23], we found that reporting UAI with 4+ non-steady partners is associated with higher age groups when compared to reporting UAI with 1-3 non-steady partners., This probably reflects that the 4+ group is rather a segment of MSM with established gay identity and having adopted a specific type of gay urban lifestyle, while the 1-3 UAI partner group represents more young MSM with a more fragile sexual identity, who are not yet integrated in the gay subculture. These two groups need probably quite different prevention approaches.

Additionally, when compared to men reporting no UAI with non-steady partners, being in a steady relationship was found to be protective, especially when men also reported being happy with their sex lives. If older men are more likely to be in steady relationships, this relationship may account for the lack of significance of age in this model. While this result is unsurprising, it is important to note that steady relationships are not necessarily free of HIV risk [24]. It is therefore crucial that public health campaigns do not ignore men with steady partners, and that a range of Safer Sex Messages for these men, including negotiated safety [25], are addressed. Furthermore, it is important to note that, among men who were not in a steady relationship, the probability of reporting 4+ partners was greater for men reporting sexual happiness in both models. Future public health campaigns could emphasize the possibility of having a full, satisfying sex life while still protecting oneself and others from HIV and other STIs.

In agreement with previous research [26, 27], we find that men who visit sex-on-site venues are more likely to report more UAI with non-steady partners. Strategies such as distribution of condoms and safe sex information at sex-on-site venues may be capable of playing a significant role in reducing UAI.

Use of nitrite inhalants, drugs associated with sex and parties, and erectile dysfunction drugs was also consistently associated with increased probability of reporting UAI with 4+ non-steady partners. The association between drug use and risky sexual behaviour has been well documented [28–30]. Drugs associated with sex and parties are typically used to enhance sociability and feelings of euphoria. Some of the newer drugs can also trigger feelings of intense sexual arousal. This crucial, additional effect has led to the use of these drugs by MSM in sexual contexts, a behaviour often referred to colloquially as ‘chemsex’ (also called ‘Party and play’), depending on the exact drugs used. The drugs are frequently combined and are typically consumed during prolonged sexual sessions, which can involve multiple sexual partners [31]. Drugs associated with sex and parties are also often combined with the more commonly used sexual performance drugs, which include both nitrite inhalants and erectile dysfunction drugs and are used to enhance and maintain erection and, in the case of nitrite inhalants, to relax the anal sphincter. Considering these contexts, the association of multiple UAI partners with use of these specific drugs is not surprising.

Our results indicate that selling and buying sex are generally associated with higher numbers of UAI partners. However, as a detailed report of the associations between transactional sex and associated health outcomes in the EMIS dataset is currently in preparation, we do not expand upon the implications of this finding here.

Additionally, we find that experiencing physical violence, and perhaps even verbal abuse and intimidation, due to sexual attraction to men was associated with reporting UAI with multiple non-steady partners. It is possible that individuals engaging in UAI with more non-steady partners may tend to visit gay sex venues more frequently, and are therefore more likely to be recognized as MSM and attacked. The more sexual partners one engages with, especially in sex-associated venues as shown in our data, the higher the likelihood to be seen outside gay venues and identified as gay by passers-by. Very few studies have previously examined the link between physical violence and HIV-related sexual risk behaviour, although Wheeler et al. found that experiencing physical violence was associated with both reporting multiple partners and STI diagnosis [32], and Santos et al. found increased risk of both UAI and HIV infection among MSM who had experienced violence due to their homo- or bisexual orientation [33]. Future research should look more closely at the association between experienced physical violence, verbal abuse, and risk behaviour.

Our consistent finding of a protective effect of higher education was in line with previous research [22]. Being exposed to information on HIV or STIs specifically designed for MSM was also associated with lower risk of reporting UAI with 4+ non-steady partners compared to no UAI partners, indicating the continuing importance of HIV education campaigns in reducing risk behaviours in this population. However, results also suggest that knowing that ART reduces HIV transmissibility is associated with higher probability of reporting UAI with 4+ non-steady partners. But this effect was very small, probably because men diagnosed with HIV and having an undetectable viral load had been excluded from our analysis. This association has been observed before, and it has been suggested that knowledge and opinions of ART are more influential than actual treatment status [34]. Still, it remains important that individuals with new HIV infections are promptly identified and given access to treatment. It should also be noted that the EMIS data were collected in 2010, and that treatment practices in many European countries likely have evolved towards earlier treatment start.

Men reporting UAI with 4+ partners were more likely to feel lonely. Meanwhile, engaging in UAI with many partners may help to temporarily reduce feelings of loneliness and enhance feelings of intimacy [35]. Thus, it may be worthwhile to invest in approaches which enable men to improve the quality of their sexual relations instead of increasing the quantity. Finally, being out to all or most of the people a participant knew was positively associated with reporting 4+ UAI partners in both models, possibly because individuals who are more out are also more comfortable seeking out sexual partners.

Rather than focusing only on whether or not an individual reports any UAI, we analyse here the factors associated with engaging in UAI with four or more non-steady partners specifically. While this makes it difficult to compare our findings to previous research, this approach is a major strength of our study in light of research showing that simple measures of “any UAI” do not accurately capture sexual risk behaviour [3, 36]. Additionally, our large dataset allowed us to consider a great number of variables in our models, and the wide geographic range of our data allowed us to analyse both individual- and country-level factors.

### Limitations

However, we also acknowledge various drawbacks of our study. Because our study was exploratory in nature and considered such a large number of variables, it is difficult to state with certainty that these variables will still be important indicators of sexual risk in other datasets or populations. Additionally, the large number of variables analysed increases the risk that some apparently significant results may actually be due to Type I errors. Furthermore, after the removal of HIV-diagnosed men with undetectable viral loads or consistent serosorting behaviour, we did not have the statistical power to ascertain how and to what extent specific variables may impact HIV diagnosed men in a different way. The change of behaviour after subjects became aware of their HIV status may not uniformly occur in the interval between the time of diagnosis and the time for interview. The analysis does not capture the dynamic changes of behaviour in different time intervals. We also note that our survey contained only very few items to assess mental health, which has been previously found to be associated with sexual risk behaviour and HIV infection [33, 37]. Due to the cross-sectional nature of our survey, we are also unable to elucidate any causal information, and temporal ambiguity bias cannot be eliminated. Finally, as with all self-reported data, there is likely to be some degree of recall bias present, and the large number of questions results in some inconsistency in responses. We also note that, since the survey was conducted online, men without internet access were unable to participate, likely leading to selection bias.

## Conclusions

In this study, we sought to identify factors independently associated with reporting UAI with four or more partners among MSM in Europe. Due to HIV testing window periods, the HIV transmission risks associated with UAI with such numbers of partners can be very challenging to manage for HIV-negative men. For men diagnosed with HIV, effective antiretroviral treatment drastically reduces HIV transmission. While increased risk of STI transmission and acquisition with multiple partners remains a concern - which can be mitigated by frequent STI testing in the context of HIV treatment monitoring - HIV transmission becomes a minor problem. The situation is different for HIV-negative men, who so far do not have access to oral HIV chemoprophylaxis in Europe. Apart from reducing partner numbers or increasing condom use no other recommendations are currently in place to reduce the risk of HIV acquisition and onward transmission for this group. More frequent testing may help to reduce onward transmission, but will not prevent acquisition of HIV.

Our results indicate that there are a variety of related factors associated with UAI with multiple partners among MSM, suggesting that reducing infection and transmission risks in this population will require a wide range of public health strategies. Such strategies should include the continued distribution of HIV-related information, as well as campaigns encouraging testing and treatment for HIV. Additionally, specific behaviours such as drug use and selling sex should receive adequate attention to ensure that men engaging in such behaviours are able to access drug and sex work-specific counselling, health promotion and prevention services to support safe sexual behaviour and reduce drug-related harms. Sex-on-site venues, as well as the Internet, are likely to be effective modes for the distribution of information for this population. Finally, it is important that MSM themselves are not the only targets of prevention campaigns; structural factors that give rise to discrimination and violence against MSM must also be combated.

### Availability of data and materials

A public use file of the EMIS dataset will be made accessible on www.emis-project.eu in the second part of 2016.
